# Infectious dermatitis associated with HTLV-I: uncommon case in southern Brazil simulating refractory atopic dermatitis^⋆^

**DOI:** 10.1016/j.abd.2020.11.017

**Published:** 2022-05-30

**Authors:** Michele Caroline dos Santos Garcia, Renata Heck, Renan Rangel Bonamigo, Cristiane Almeida Soares Cattani

**Affiliations:** a Ambulatório de Dermatologia Sanitária do Rio Grande do Sul, Porto Alegre, RS, Brazil; b Faculty of Medicine, Universidade Federal do Rio Grande do Sul, Porto Alegre, RS, Brazil

Dear Editor,

HTLV-I (human T lymphotropic virus type-I), a human retrovirus discovered in the 1980s,^1^ infects preferentially CD4 T lymphocytes. The worldwide prevalence is uncertain, with an estimated 5 to 10 million infected individuals,^2^ mainly in Japan, Iran, Latin America, and Africa.^3^, ^4^

Infectious dermatitis associated with HTLV-I (IDH) was described in Jamaica in 1966, and associated with HTLV-I in 1990, being a rare and treatment-resistant form of exudative dermatitis.^1^, ^3^, ^4^, ^5^

We describe a case of a seven-year-old girl, from the south of Brazil, born through vaginal delivery, with severe recurrent eczema since she was 18 months of age, when she stopped being breastfed.

On examination, she had macerated, exudative, and foul-smelling eczematous lesions on the scalp and retroauricular, cervical, antecubital, and intergluteal regions; temporal alopecia; crusts in the umbilical, perioral and nasal regions (Figure 1, Figure 2). Laboratory tests were normal, except serology for HTLV-I/II which was reactive, confirming the diagnosis of IDH according to the criteria described in Table 1.^5^ The other viral serologies were negative. The neurological examination was normal. Her mother also had positive serology for HTLV-I/II. Treatment with oral sulfamethoxazole and trimethoprim was started, with significant clinical improvement.

**Figure 1:**
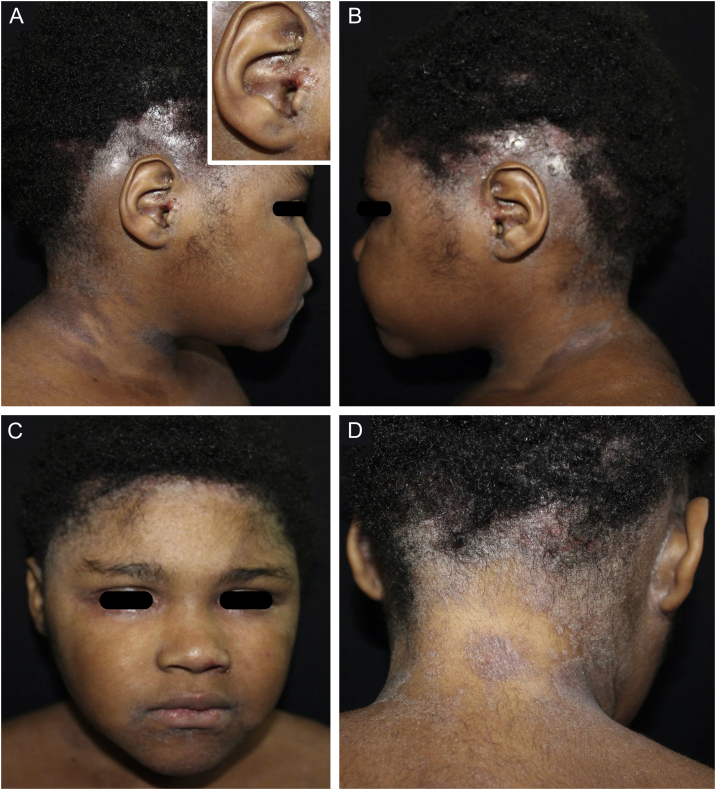
(A–D), Eczematous and exudative lesions on the scalp, with areas of alopecia, and excoriations in the external auditory canal. In A, a detail of the affected external auditory canal. C, infrapalpebral erythematoedematous areas and perilabial eczematous lesions. D, eczematous lesions in the cervical and retroauricular regions; occipital alopecia.

**Figure 2:**
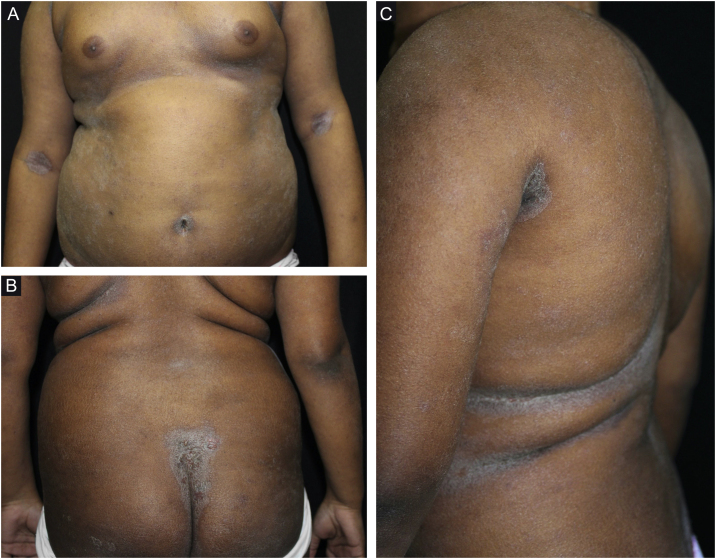
(A–C), Eczematous lesions on the trunk, antecubital fossae and intergluteal region, associated with diffuse xerosis.

**Table 1:** Major criteria for the diagnosis of infectious dermatitis associated with HTLV-I.

1. Presence of erythematous-desquamative, exudative and crusted lesions on the scalp, retroauricular areas, cervical and inguinal regions, axillae, perioral and paranasal skin, ears, chest, abdomen and other sites
2. Crusts on the nostrils
3. Chronic recurrent dermatitis with immediate response to appropriate therapy, but recurrence soon after discontinuation of antibiotics
4. Diagnosis of HTLV-I infection by serological or molecular biology tests

Adapted from La Grenade^5^ et al. apud De Oliveira et al.^1^

Of the four main criteria, three are necessary for the diagnosis; 1, 3 and 4 are mandatory.

Criterion 1 requires the involvement of three or more sites, including the scalp and retroauricular areas.

IDH usually starts in childhood and is considered an early clinical marker of HTLV-I infection.^3^, ^4^ The main route of transmission is through breastfeeding.^3^, ^5^ Its pathogenesis involves individual susceptibility, immune dysregulation, bacterial superinfection, environmental antigenic stimulation and persistent inflammation.^4^ The pro-inflammatory state may be related to the proliferation of T lymphocytes and high levels of IL-1, IL-6, TNFα and IFNα; elevated IgE levels increase susceptibility to *S. aureus* and *S. beta-haemolyticus*.^4^

Patients should be screened for HTLV-I in cases of severe, resistant, recurrent eczema with secondary infection.^4^ Atopic dermatitis (AD) and seborrheic dermatitis are the main differential diagnoses.^4^ Histopathology is non-specific and CD8 T lymphocytes predominate in immunohistochemistry.^4^ Approximately 10% of those infected develop adult T-cell leukemia/lymphoma and HTLV-I-associated myelopathy/adult tropical spastic paraparesis.^2^, ^3^, ^4^ Symptoms tend to show remission at puberty but persist if they start at the adult age.^1^, ^4^

IDH does not have a specific treatment or vaccine; however, it usually responds to antibiotics such as sulfamethoxazole and trimethoprim, and cephalexin, for long periods, with recurrence being common.^3^, ^4^ Infected individuals must be monitored due to the possibility of severe neurological and lymphoproliferative complications.

The interruption of the transmission involves screening blood donors, using condoms, family counseling, avoiding breastfeeding, and avoid sharing needles.^4^

IDH is relevant in the practices of dermatologists, infectologists, hematologists and neurologists and, despite its absence from the lists of neglected diseases, the perception is that it is very close to that situation.^4^ It is not compulsorily notified, and there are not even policies for the prevention or care for the virus carriers.

We emphasize the importance of this case, as it occurred outside the endemic areas in Brazil – which are the northern and northeastern regions ‒ and because it was managed as a recalcitrant AD for a long period.

## Financial support

None declared.

## Authors' contributions

Michele Caroline dos Santos Garcia: Design and planning of the study; drafting and editing of the manuscript; collection, analysis, and interpretation of data; intellectual participation in the propaedeutic and/or therapeutic conduct of the studied cases; critical review of the literature; critical review of the manuscript; approval of the final version of the manuscript.

Renata Heck: Critical review of the manuscript; intellectual participation in the propaedeutic and/or therapeutic conduct of the studied cases; effective participation in research orientation; approval of the final version of the manuscript.

Renan Rangel Bonamigo: Critical review of the manuscript; intellectual participation in the propaedeutic and/or therapeutic conduct of the studied cases; effective participation in research orientation; approval of the final version of the manuscript.

Cristiane Almeida Soares Cattani: Design and planning of the study; critical review of the manuscript; intellectual participation in the propaedeutic and/or therapeutic conduct of the studied cases; effective participation in research orientation; approval of the final version of the manuscript.

## Conflicts of interest

None declared.
